# Original quantitative research - Accidental substance-related acute toxicity deaths among youth in Canada: a descriptive analysis of a national chart review study of coroner and medical examiner data

**DOI:** 10.24095/hpcdp.44.3.02

**Published:** 2024-03

**Authors:** Grace Yi-Shin Chang, Amanda VanSteelandt, Katherine McKenzie, Fiona Kouyoumdjian

**Affiliations:** 1 Public Health Agency of Canada, Ottawa, Ontario, Canada; 2 Ontario Ministry of Health, Toronto, Ontario, Canada

**Keywords:** substance use, drug overdose, opioid overdose, acute toxicity deaths, children, youth, young adults, Canada

## Abstract

**Introduction:**

Substance-related acute toxicity deaths (ATDs) are a public health crisis in Canada. Youth are often at higher risk for substance use due to social, environmental and structural factors. The objectives of this study were to understand the characteristics of youth (aged 12–24 years) dying of accidental acute toxicity in Canada and examine the substances contributing to and circumstances surrounding youth ATDs.

**Methods:**

Data from a national chart review study of coroner and medical examiner data on ATDs that occurred in Canada between 2016 and 2017 were used to conduct descriptive analyses with proportions, mortality rates and proportionate mortality rates. Where possible, youth in the chart review study were compared with youth in the general population and youth who died of all causes, using census data.

**Results:**

Of the 732 youth who died of accidental acute toxicity in 2016–2017, most (94%) were aged 18 to 24 years. Youth aged 20 to 24 who were unemployed, unhoused or living in collective housing were overrepresented among accidental ATDs. Many of the youth aged 12 to 24 who died of accidental acute toxicity had a documented history of substance use. Fentanyl, cocaine and methamphetamine were the most common substances contributing to death, and 38% of the deaths were witnessed or potentially witnessed.

**Conclusion:**

The findings of this study point to the need for early prevention and harm reduction strategies and programs that address mental health, exposure to trauma, unemployment and housing instability to reduce the harms of substance use on Canadian youth.

**Highlights**

- In 2016 and 2017, nearly half (46%) of all accidental deaths among youth 18 to 24 years of age were due to acute toxicity.
- Youth aged 20 to 24 who were unemployed, living in collective housing or unhoused were overrepresented among those who died of accidental acute toxicity.
- Almost one-third (30%) of youth 12 to 24 years of age who died of accidental acute toxicity had at least one documented potentially traumatic event during their life.
- Opioids (fentanyl, morphine, diacetylmorphine [heroin], carfentanil) and stimulants (cocaine, methamphetamine, amphetamine) of nonpharmaceutical origin were the most common contributors to accidental acute toxicity deaths among youth aged 12 to 24 years.
- Thirty-eight percent of the accidental acute toxicity deaths in youth were witnessed or potentially witnessed.

## Introduction

Substance-related acute toxicity deaths (ATDs) are a public health crisis in Canada that have had a serious impact on youth. Between 2013 and 2017, there was a 53% increase in rates of opioid poisoning–related hospitalizations of youth aged 15 to 24.^1^ In March 2022, pediatricians reported seeing a concerning number of children and youth with severe or life-threatening cases of opioid, stimulant or sedative use in the previous 24 months.^2^ Youth face unique social, environmental and structural factors that contribute to substance use and can lead to poor overall health, mental health conditions and death.^3^,^4^

As the risk of ATD is often higher for youth with a history of substance use or substance use disorders, it is critical to explore the risk factors of substance use and substance use disorders in order to understand the risk of ATD.^5^,^6^ Adolescents’ risk factors for substance use and substance use disorders are unique to this age group due to the many changes that come with this transitional period of life.^5^ Some risk factors include adverse childhood experiences (such as abuse, traumatic events, neglect and mental health conditions in family members),^3^,^4^ mental health conditions,^7^-^9^ history of correctional involvement^10^ and family history of substance use.^3^,^4 ^

Analyses of death investigation data from Ontario and British Columbia and linkage of census and vital statistics data have revealed factors specifically associated with ATDs among Canadian youth.^6^,^11^-^12^ These factors include neighbourhood-level income inequality,^11^ living arrangements and housing instability, the absence of a bystander who could intervene, a mental health diagnosis^6^,^12^ and receipt of current or previous child, youth or family services.^12^

Previous research has highlighted challenges in opioid-related services for youth, including gaps in the continuum of care, inaccessibility of services, stigma, lack of respect for youth autonomy and a lack of family supports.^13^-^16^ Opioid agonist therapy prescription rates and residential treatment rates have been declining among Ontario youth since 2014, despite increasing opioid-related youth ATDs.^6^ About half of the youth who died from opioid-related acute toxicity in Ontario had an opioid use disorder. Difficulties youth face in accessing treatment or harm reduction services that suit their needs and preferences limit their protection from an increasingly toxic and unpredictable illegal drug supply. Nonpharmaceutical fentanyl has been the primary contributor to youth ATDs in Ontario and British Columbia in recent years.^6^,^12^ Youth who use substances intermittently may be at particular risk of opioid toxicity because they have less experience and lower opioid tolerance.^17^

Previous research has explored ATDs within provinces or cities or with a focus on subpopulations of youth, but only a few studies have examined ATDs among youth at a national level in Canada.^6^,^11^-^12^,^18^-^21^ The purpose of this study was to address these knowledge gaps by examining ATDs among youth based on Canadian death investigation data from 2016 and 2017, and to set a baseline in the early years of the overdose crisis for comparison with future research. The objectives of this study were

- (1) to report the minimum prevalence of risk factors for substance use and substance use disorders identified by previous research among youth who died of accidental acute toxicity in Canada in 2016 and 2017;
- (2) to examine the most common substances contributing to ATDs among youth; and
- (3) to describe the circumstances of ATDs among youth.

## Methods

***Ethics approval***

This study was reviewed and approved by the Public Health Agency of Canada Research Ethics Board (REB 2018-027P), the University of Manitoba Health Research Ethics Board (HS22710) and Newfoundland and Labrador Health Research Ethics Board (20200153).

***Data sources***

This analysis uses data on 732 accidental ATDs of youth taken from a retrospective chart review study of coroner and medical examiner death investigations of ATDs in all Canadian provinces and territories that occurred between 1 January 2016 and 31 December 2017.^22^ An ATD is defined as a death after an acute toxicity due to the direct effects of one or more drugs or alcohol.^23^ Further details on the study protocol and the variables collected have been published elsewhere.^22^ Using census data from 2016^24^-^27^ and Canadian Vital Statistics- Death data from 2016 and 2017^28^ permitted comparisons to the general population and the calculation of mortality rates. 

***Youth definition***

In this study, youth are defined as individuals 12 to 24 years of age. To capture the differences among youth within this age range, this study stratifies youth into two categories: those between 12 and 17years of age and those between 18 and 24 years of age. Each group has unique characteristics, and some variables are more age-dependent than others. Youth between 12 and 17 years of age are most likely students who live with parents or guardians, whereas youth aged 18 years and older may be legally permitted to use some substances and may no longer live with a parent or guardian and may have increased independence. 

While youth aged 12 years and older are more likely to be actively using substances, children younger than 12 are more likely to experience accidental exposure to substances. Given the difference in the type of exposure (unintentional use is a different phenomenon from intentional use of substances) and the small number of ATDs in this age group, children under 12 were excluded from this study. Most of the analysis in this study includes youth aged between 12 and 24. However, to compare with the 2016 Census data, results for those aged 20 to 24 years are presented separately.

***Variables of interest***

The primary outcome variable in this analysis was ATDs. The chart review study dataset provided data on previously identified risk factors for substance use, substance use disorders and ATDs among youth. These include sociodemographic factors (i.e. age, sex, employment status, living arrangement) and social or medical history (i.e. history of incarceration, contact with health services in the preceding year, history of mental health conditions or symptoms, history of substance use, history of substance use disorder and potentially traumatic life events). 

Contact with health services includes inpatient admission (hospital or other) and outpatient treatment (e.g. emergency medical services, emergency department, general practitioner or nurse practitioner). 

Potentially traumatic events are used as the best comparable measure to adverse childhood events from the death investigation files. Potentially traumatic events include one-off events, series of events or circumstances that are physically or emotionally harmful or life threatening and could have lasting adverse effects on the person’s mental, physical, social, emotional or spiritual well-being.^29^ They might include a health problem of a family member or relative, intimate partner problems, other relationship problems (e.g. a family argument), job or school problems, financial problems, the recent death of a friend or family member, criminal or other legal problems (e.g. custody dispute, civil suit), perpetrating or being a victim of interpersonal violence or a victim of child abuse, foster care experiences, experiencing sexual abuse, or experiencing physical abuse or assault. 

These variables were collected from any available source in the death investigation file, which might include statements from family, friends or a primary health care provider, medical records, autopsy reports or police reports, for example. Therefore, some of the medical conditions reported may not necessarily have been medically diagnosed. Where available, residential postal codes were linked to Statistics Canada’s Postal Code Conversion File Plus to obtain area-based neighbourhood income quintile after tax (QAATIPPE).^30^

In this paper, the substances contributing to death are reported by their origin (pharmaceutical or nonpharmaceutical) and whether the substance contributed to death alone or in combination with other substances. 

Variables that indicate the circumstances of the acute toxicity events and death include the most likely mode of substance use, the presence of a witness, the actions taken by the witness during the first and subsequent encounters, the administration of naloxone for youth with symptoms of opioid toxicity, the place of the acute toxicity event and the place of death. We also examined whether the place of the acute toxicity event was indoors or outdoors, whether the person was found in or near a bed or in a vehicle and, in the case of youth who experienced the acute toxicity event inside their personal residence, whether they lived alone or with someone else. 

***Statistical methods***

To calculate accidental acute toxicity mortality rates and proportionate mortality due to acute toxicity, we used population data from the 2016 Census and all-cause accidental death counts from the Canadian Vital Statistics - Deaths Database as denominators.^24^,^28^ As a person’s entire life history is not documented in coroner and medical examiner files and there is variation in what is collected across jurisdictions, there is likely additional history and information that was not captured. The results of this study should therefore be considered the minimum proportions of youth that had a given characteristic. Census data were used to compare proportions and calculate mortality rates for youth aged 20 to 24 by employment status and living arrangement.^25^-^27^ For the remainder of the analyses, the minimum proportions of youth aged 12 to 24 who had a given characteristic were calculated. An UpSet plot was constructed using the ComplexUpSet package to identify the most common substances and substance combinations contributing to death, and their origin.^31^

All statistical analyses were performed using R statistical software version 4.1.1.^32^ To protect privacy, cell sizes less than 10 were either suppressed or grouped into larger categories, all counts were randomly rounded to base 3 and proportions and mortality rates were based on rounded counts.^22^

## Results

***Burden of acute toxicity deaths among youth***

Overall, there were 732 people aged 12 to 24 years who died of accidental acute toxicity, and most of these youth were between 18 and 24 years of age (94%; Table 1). Death due to accidental acute toxicity accounted for nearly half (46%) of all-cause accidental mortality among youth aged 18 to 24. Among youth aged 12 to 17 years, the contribution of acute toxicity to all-cause accidental deaths was higher among females (23%) than males (11%). 

**Table 1:** Number of deaths, mortality rates and proportionate mortality rates for youth aged 12 to 24 years in Canada who died from accidental substance-related acute toxicity in 2016 and 2017

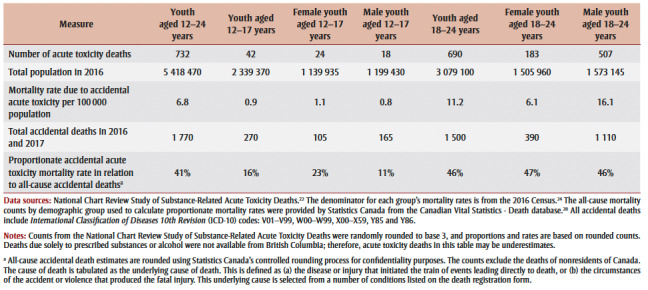

***Characteristics of youth who died of accidental acute toxicity***

A subset of 567 youth between 20 and 24years of age who died of accidental acute toxicity in 2016 or 2017 were compared to youth of the same age using 2016 Census data (Table 2). The employment status of youth who died of acute toxicity was most often unknown (49%), and the other employment status categories in Table 2 represent the minimum proportions of youth in that category. At least 23% of individuals who died were employed and at least 18% were unemployed. Given the number of unknowns, the employment rate among those who died of acute toxicity may be lower, equivalent or higher than the rate in the general population. However, unemployment was more prevalent among youth who died (at least 18% but perhaps higher) than among youth in the general population (11%). The mortality rate due to acute toxicity for youth who were unemployed was 20.4 per 100000. 

**Table 2:** Comparison of employment status and living arrangements for youth aged 20 to 24 years who died of accidental acute toxicity (2016 to 2017) and in the Canadian general population (2016)

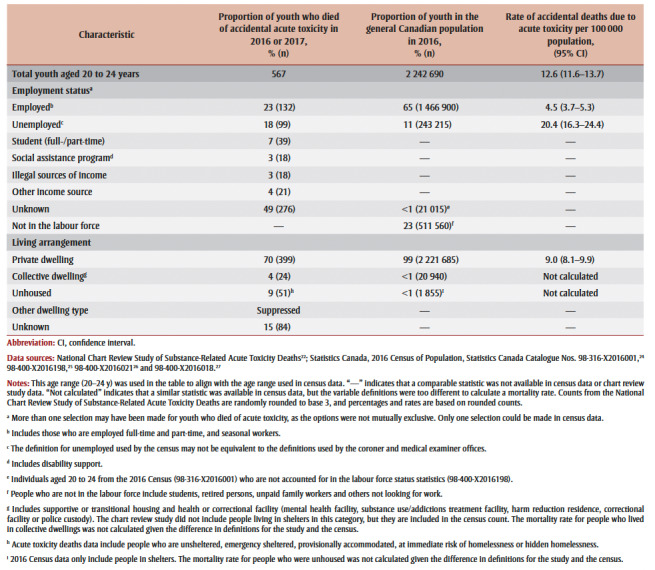

While most youth aged 20 to 24 lived in a private dwelling (70%), 4% lived in a collective dwelling and 9% were unhoused at the time of their death. Youth who were living in collective dwellings or unhoused were overrepresented among those who died of acute toxicity when compared to the general population. We did not calculate mortality rates for these two groups due to differences in the definitions used by the two data sources. 

Table 3 presents the characteristics of the 732 youth aged 12 to 24 years who died of accidental acute toxicity in 2016 and 2017. Commonly documented mental health conditions or symptoms in this group included depressive disorder or depressive symptoms (22%), substance use disorder (excluding alcohol; 20%), anxiety disorder (16%) and suicidal ideation or suicide attempt (12%). Eighty-three percent of youth had a documented history of substance use, and more than half (59%) had contact with health care services in the year preceding their death. At least 30% of the youth had experienced a potentially traumatic event in their lifetime, the most common of these being a criminal legal problem (e.g. arrest, jail time, court hearing; 14%). About one in 20 (5%) experienced a potentially traumatic event in the two weeks before their death. Youth from the lowest neighbourhood income quintile were overrepresented among those who died. It is likely that many of the youths with unknown residential postal codes also belonged to lower income quintiles, because at least one-quarter of those with unknown postal codes were unhoused at the time of their death (data not shown).

**Table 3:** Characteristics of youth aged 12 to 24 years who died of accidental acute toxicity in Canada, 2016 or 2017

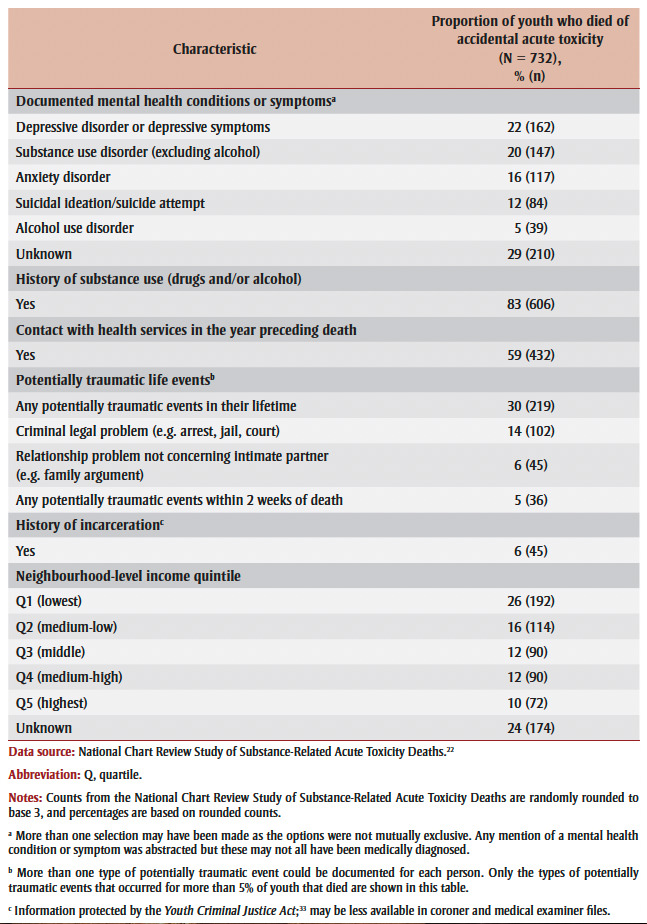

***Substances contributing to accidental ATDs***

Fentanyl (56%), cocaine (30%), methamphetamine (18%) and ethanol (alcohol; 16%) were the most common substances identified as contributing to death among youth who died by accident (Table 4). Seven of the substances contributing to at least 5% of deaths were opioids (fentanyl, morphine, diacetylmorphine [heroin], carfentanil, methadone, oxycodone and hydromorphone); four were stimulants (cocaine, methamphetamine, amphetamine and MDMA); and the other two were alcohol and a benzodiazepine (alprazolam). The substances contributing to accidental deaths for youth were most often of nonpharmaceutical origin. Carfentanil, methadone, fentanyl, cocaine and ethanol (alcohol) contributed to deaths on their own (without the contribution of other substances) more often than other substances.

**Table 4:** Origin of the most common substances contributing to accidental acute toxicity deaths among youth 12 to 24 years of age in Canada, 2016 to 2017

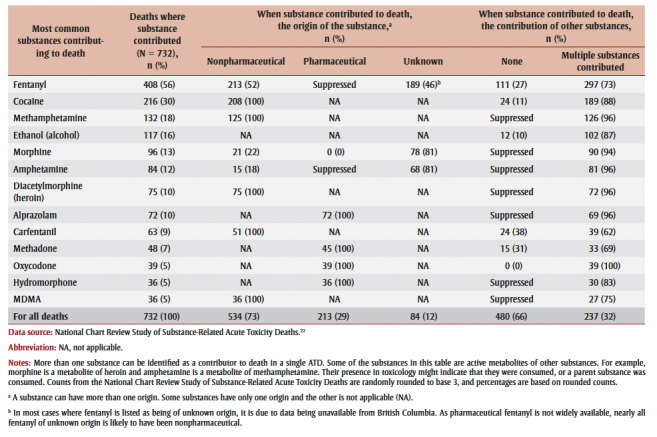

**Figure 1:**
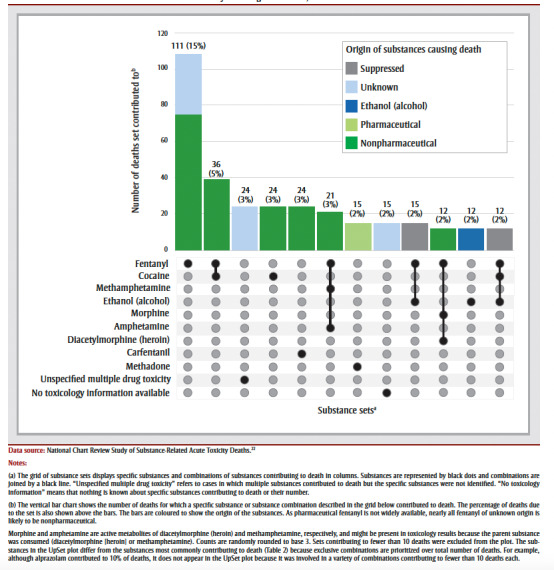
UpSet plot of the most frequent substances and substance combinations contributing to accidental deaths for youth 12 to 24 years of age in Canada, 2016 to 2017

The substances and substance combinations that were the most common contributors to accidental deaths among youth 12 to 24 years of age were fentanyl alone (111deaths, 15% of youth) and fentanyl and cocaine in combination (36 deaths, 5% of youth; Figure 1). Most of the top substances and combinations involved opioids and/or stimulants, and most were drugs of nonpharmaceutical origin.

***Circumstances of accidental acute toxicity event and death***

During the acute toxicity event that led to death, the mode of substance use by youth aged 12 to 24 was most often unknown; however, similar proportions of youth were likely using substances orally (15%), through nasal insufflation (snorting; 14%), smoking (13%) or injection (11%; Table 5). 

**Table 5:** Circumstances of death for accidental acute toxicity events and deaths among youth 12 to 24 years of age in Canada, 2016 to 2017

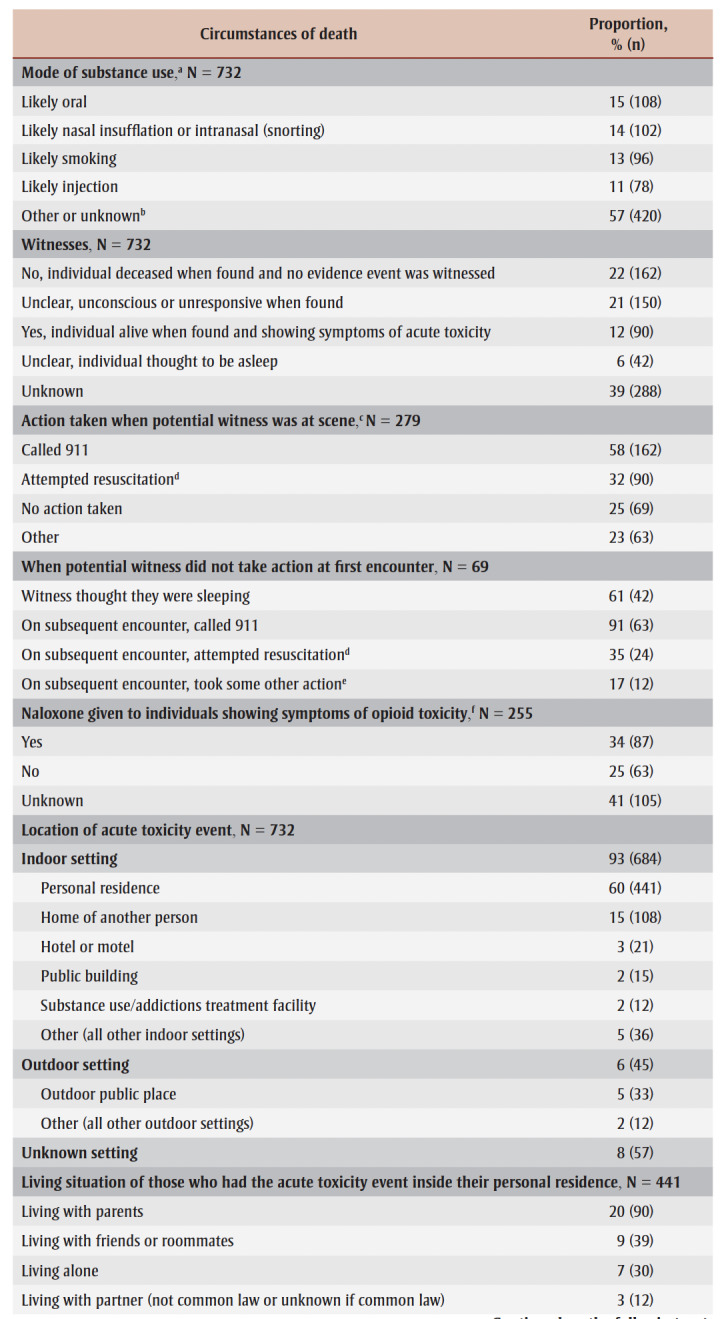 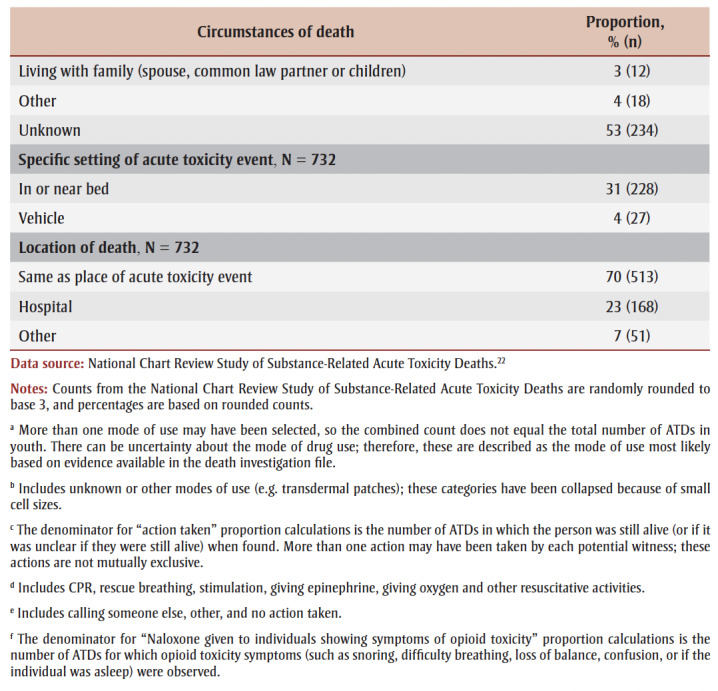

For 38% (279) of youth, the acute toxicity event was witnessed or potentially witnessed (the person was still alive when found, or it was unclear) (Table 5). In these situations, witnesses called 911 58% of the time and attempted resuscitation 32% of the time. No action was taken by potential witnesses for one in four acute toxicity events (25%). When no action was taken at the first encounter, the potential witnesses believed the individual was sleeping 61% of the time. Other reasons the potential witness might not have taken action at the first encounter included not recognizing a medical emergency or lack of access to a phone, for example. 

During subsequent encounters, potential witnesses called 911 in 91% of cases and attempted resuscitation in 35% of cases. When individuals showed symptoms of opioid toxicity (such as snoring or gurgling, difficulty breathing, pinpoint pupils, being unconscious or unresponsive, or blue lips, fingernails or face), naloxone was given at least 34% of the time. 

The most common place of acute toxicity event was in a personal residence setting (60%) followed by the home of another person (15%). A minority of acute toxicity events occurred outside (6%) or inside a vehicle (4%). Almost one-third of those who died were found in or near a bed (31%). Most youth died where the acute toxicity event occurred (70%), while 23% died in a hospital.

## Discussion

The findings of this study highlight the minimum prevalence of factors among accidental youth ATDs that previous researchers have identified as important for youth substance use, substance use disorders, and ATDs. Most youth who died of acute toxicity had a history of substance use (83%), and 20% had a documented history of a substance use disorder. Mental health conditions or symptoms such as depressive disorder or depressive symptoms, anxiety disorder and suicidal ideation or suicide attempts were commonly documented among youth who died of acute toxicity. 

Previous research has pointed to the relationship between mental illness, history of substance use and substance use disorders;^5^-^6^,^12^ this study’s findings further illuminate the need for early prevention, treatment and harm reduction for substance use and accessible mental health care and supports for youth to prevent ATDs. More than half of youth had contact with health services in the year preceding their death, suggesting a potential opportunity for intervention. Ensuring that substance use services for youth are appropriately tailored to the unique needs of this population is important. Improved and targeted supports for youth, including during transitions to early adulthood, could reduce harms in this population.

Additionally, about one-third of youth who died of accidental acute toxicity experienced at least one potentially traumatic event in their lifetime. This finding aligns with existing literature on the role that trauma plays in substance use disorders.^34^ However, different types of traumatic events may impact individuals differently, and may or may not always be related to substance use. Death investigations do not deliberately set out to collect potentially traumatic events that occurred earlier in life; therefore, the number of youth who had been exposed to traumatic events is likely higher than was documented in this study. In British Columbia, 73% of youth aged 18 and younger who died of an unregulated drug-related acute toxicity were in receipt of child, youth or family services.^12^

The results of this study also suggest potential opportunities for targeted upstream interventions. Male youth aged 18 to 24 accounted for the highest proportion of youth ATDs. Unemployed youth had a very high mortality rate due to acute toxicity, though it is unknown if all people described as unemployed in their coroner or medical examiner file met the Statistics Canada definition. Youth who lived in collective housing or were unhoused were overrepresented among those who died of acute toxicity. The Statistics Canada definition of collective housing includes more institution types than that used by the chart review study, yet the minimum proportion of youth who died of accidental acute toxicity and lived in collective housing was higher than that of the general population of youth (4% and <1%, respectively). The Statistics Canada definition of being unhoused counted only those who were staying in a shelter, while the chart review study definition also included youth unsheltered on the street or temporarily staying with family or friends. Nevertheless, it is very concerning that nearly 1 in 10 youth who died of accidental acute toxicity were documented as unhoused—a situation that should be rare. 

Developing prevention and harm reduction programs specifically for youth who are unemployed, living in collective housing or unhoused would reach a large proportion of youth at risk for ATD. These findings also highlight a need for improved service integration for this population, so that other social supports such as housing and employment that are often interrelated are easily accessible alongside mental health and substance use services. 

The substances most commonly contributing to youth ATDs were opioids and stimulants of nonpharmaceutical origin. In Canada, illegal fentanyl was first recognized in 2011, and by 2016, opioids were among the top 10 controlled substances most detected by Canada’s Drug Analysis Service (DAS).^35^ Additionally, over half of the heroin samples tested by DAS between 2012 and 2017 contained fentanyl.^35^ As youth may not have been aware of these changes in the drug supply, increasing awareness of the presence of fentanyl in other substances could potentially reduce accidental ATDs.^4^,^36^ People who have been using opioids for a long time develop a tolerance, requiring greater amounts over time to achieve the same effects.^17^ Those who have just started using substances or who have had a break in substance use—situations in which many youth may find themselves—cannot tolerate greater amounts, and highly toxic opioids such as fentanyl present a greater risk. 

Evidence-based prevention and treatment for opioid toxicity such as naloxone access, opioid agonist treatment and supervised consumption sites are key.^37^ Drug-checking services that are accessible to youth may allow them to test that the substances they purchase on the street are not contaminated with dangerous drugs.^37^ Additionally, services that offer prescribed medications as an alternative to illegal drugs may give youth access to a safer supply while connecting them with health and social services.^38^

Another important method to reduce the risk of an ATD is to not use substances alone.^17^ Only 38% of accidental acute toxicity events leading to death among youth were witnessed or potentially witnessed by someone who could call for help or give aid. Among the youth whose fatal acute toxicity event occurred in their personal residence, 20% lived with their parents, 9% lived with friends or roommates and only 7% were living alone. For many of those who died, someone might have been nearby while they used substances, but stigma may have prevented the youth from telling others about their substance use and having someone who could support them in the event of an emergency.^39^

The ability of the bystander to recognize and respond to an acute toxicity event is also important. About one in three youth were found in or near a bed, and when bystander actions were delayed, it was most often due to a belief that the person was asleep or sleeping off substance use. These findings suggest a need for increasing awareness of the signs of acute toxicity and how it may be confused with sleep so that bystanders can better recognize the medical emergency. 

Other reasons bystanders might not act immediately could be the absence of a phone to call 911 or not having naloxone on hand, for example. Improving access to naloxone and the means of calling emergency health services would increase bystanders’ ability to act more rapidly. 

Targeted public education and awareness efforts on the toxicity of the drug supply and recognizing and responding to an overdose, as well as to reduce stigma around substance use in this population remain important. It is also relevant to note that the study period reflects the early stages of the overdose crisis before communications and messaging efforts were widely increased. 

Some of the youth in this study were above the legal threshold for majority, and some were below; there are big differences in how laws, policies and practices are applied to these two groups.^36^ While minors are often grouped together in these analyses due to small counts, interventions for youth under the age of 18 must consider the roles of parents and guardians and children’s rights.

***Strengths and limitations***

This study provides an important baseline to examine ATDs among Canadian youth on the national level near the beginning of the overdose crisis. Coroner and medical examiner data provide details on the circumstances of death, such as the location of death and the presence of witnesses, and are often more comprehensive than other mortality data sources. 

However, it is important to note that death investigations are not methodologically designed to collect all variables of interest to our study, and some variables may be more likely to have missing data than others. Information protected by the *Youth Criminal Justice Act*,^33^ such as history of incarceration, may have been less available in the death investigation files for youth. Based on the numbers reported by British Columbia,^12^ death investigation files provide limited documentation of child, youth or family services received by youth who died. Therefore, as information is missing for many of our variables of interest, the findings in this analysis only represent the minimum proportions of youth characteristics, and the mortality rates and proportions presented are likely underestimated. 

Both the drug supply and environmental stressors have changed since the study period in 2016 and 2017, particularly during the years of the COVID-19 pandemic. Some of the findings may no longer be as relevant to youth today, as the substances contributing to death and the harm reduction practices adopted by youth may have changed since 2017. There has been conflicting evidence on whether substance use among youth decreased or increased during the COVID-19 pandemic; changes in the patterns of substance use may be due to other factors.^40^-^43^

Based on an Ontario comparison of youth aged 15 to 24 who died of opioid-related acute toxicity during the pre-pandemic period versus the pandemic period, we might expect to see an increase in the proportion of deaths to which nonpharmaceutical fentanyl or benzodiazepines contributed, fewer deaths outdoors and an increase in substance use via inhalation or smoking in recent years across Canada after our study period.^6^ However, as the pace of change and context of the overdose crisis varies across provinces and territories, it is difficult to extrapolate from one province to the national level. 

Nevertheless, despite the older study period, this study identified concerning upstream factors that were highly prevalent among youth who died of acute toxicity and can serve as a baseline for future studies.

## Conclusion

This study provides an important baseline near the beginning of the overdose crisis for examining ATDs among Canadian youth on the national level, and will support future work investigating how the crisis has evolved over time. Understanding the characteristics of youth who died of accidental acute toxicity, the substances contributing to their deaths and the circumstances of death can inform harm reduction and social programs and policies to better meet the needs of youth and prevent further acute toxicity deaths. Additionally, these findings highlight the need to implement early prevention interventions that address mental health, exposure to trauma, unemployment and homelessness to reduce the harms of substance use on Canadian youth. 

## Acknowledgements

We would like to acknowledge our collaborators at the offices of chief coroners and chief medical examiners across Canada for providing access to their death investigation files, and Jenny Rotondo, Brandi Abele, Songul Bozat-Emre, Matthew Bowes, Jessica Halverson, Dirk Huyer, Beth Jackson, Graham Jones, Jennifer Leason, Regan Murray, Erin Rees and Emily Schleihauf for their contributions to developing the National Chart Review Study of Substance-Related Acute Toxicity Deaths. 

## Conflicts of interest

None.

## Authors’ contributions and statement

YSC, AV, KM, FK: Conceptualization.

YSC, AV: Analysis.

YSC: Writing—original draft.

YSC, AV, KM, FK: Writing—review & editing.

YSC, AV: Project administration.

AV: Supervision. 

The content and views expressed in this article are those of the authors and do not necessarily reflect those of the Government of Canada. This report is based on data and information compiled and provided by the offices of chief coroners and chief medical examiners across Canada. However, the analyses, conclusions, opinions and statements expressed herein are those of the authors, and not necessarily those of the data providers.
